# New Drugs, Appliances, and Things Medical

**Published:** 1891-12-12

**Authors:** 


### NEW DRUGS, APPLIANCES, AND THINGS MEDICAL.

[All preparations' appliances, novelties, etc., of which a notice is desired, should be sent for The Editor, to care of The Manager, 140, Strand, London, W.C.]

### AT THE BRITISH MEDICAL ASSOCIATION.

(*Concluded from page* 11.)

H. K. Lewis, 136, Gower Street, W.C., had a table-full of his most recent publications for the medical world, arranged in a tasteful and pleasing manner.

Arnold and Sons, West Smithfield, E.C., besides a good collection of the usual surgical exhibits, had a long series of inventions of the most recent type. These were Dr. Duke's of Dublin: Needle carrier, breast support, clamp scissors, vaginal insufflator, boric acid intrauterine tube, rapid dilator, and double uterine dilator. The Howard physician's and surgeon's chair and useful consulting room adjunct. A new form of perineal curve midwifery forceps by Dr. Pearse; and numerous other novelties.

In the entrance lobby, Caleb Loader, Wincanton, Somerset, and J. Gillingham, Chard, Somerset, exhibited well made spinal and reclining chairs. The former had an ingenious arrangement in invalid chairs and beds called the "equipoise." Both inventors' productions were marked by great taste and neatness of make.

Section E.

Pocock Bros., 235, Southwark Bridge Road, had on view their well-known combined mattress and air and water bed, and also the Universal Invalid Tubular Water and Air Bed. This, by being made in sections, admits of ventilation, regulation for pressure on a part, adaptability to any angle required. There were good models of a padded room for asylums, and very ingenious epileptic bed and bedstead.

We next come to the old-established firm of bootmakers, Dowie and Marshall, 454, West Strand, with a display of perfect and very imperfect feet, and the various methods by which they fit and render comfortable both orders of supporters. As an easy shoe is one of the geatest comforts in life, we do not wonder at the somewhat remarkable collection of autographs possessed by this firm from customers of all ranks.

The Leather and Rubber Company (Limited), Leeds, made a varied exhibit of their "Hygiene" boots, which are constructed for every occasion for which a boot is required There were some excellent specimens of boots for medical men and for hospital use, besides boots and shoes for all kinds of sports, from shooting to tennis.

Billington Brothers, Abercromby Works, Cambridge Street, Liverpool, had on view a great variety of beds for as great a variety of purposes. We specially noticed a "Patent Asylum" bedstead, with the wire mattress so attached as to prevent the patient getting between the spring mattress and the frame of the bedstead.

George Gale and Sons, 32, Brooke Street, Holborn, E.C., also showed bedsteads and mattresses. We have commented favourably on these in our columns, and again state our impression that the "Lawson Tait" hospital combination bedstead is one of, if not the best, bedsteads for hospital purposes that we have met with.

Taylors (Limited), 9, Leet Street, Liverpool, showed their automatic disinfector, by the use of which the drains can be disinfected 3,000 times for 5s.

John Greenall, 120, Portland Street, Manchester, exhibited his steam washer, which is suitable for private houses as well as public institutions. By its use rubbing and scrubbing are done away with, and as the clothes are subjected to the action of steam, they are disinfected as well as washed.

The Water-Softening Company (Limited), 11, Delahay Street, S.W., showed their domestic apparatus for softening water under pressure; also plans of this and Carrod's non-pressure automatic water-softener.

Goddard, Massey, and Warner, Nottingham, exhibited plans of their super heated steam and hot-air disinfector. This not only steams the infected clothes at a great heat, but also by means of an ingenious arrangement keeps them dry by the use of heated air. The same firm also had plans of their Perfectus destructor as in use at Bournemouth, Hornsey, and Newcastle-on-Tyne. These can, by proper dampers, be adapted to destroy any kind of refuse, and yet prevent nuisance from smell or fine dust.

The Atkins Filter and Engineering Company (Limited), 65, Farringdon Road, E.C., the Government contractors. The filtering material consists of pure charcoal blocks, and as they can be thoroughly inspected and cleaned without sending to the makers, we do not wonder that both the Royal Colleges and over thirty of the London hospitals use them.

Washington Lyon's patent steam disinfector. It is claimed that all forms of insect life are immediately destroyed, and that a moist heat up to 260° F. can be obtained without any risk of burning garments submitted to the action of the apparatus. The heat is regulated simply by attending to the steam pressure.

Coghlan McHardy, 1, Grenville Place, Cromwell Road, S.W. Improved patented carrying chair. It is so constructed that going upstairs, downstairs, or on the level, the seat always remains in the horizontal position irrespective of the angle at which the handles may be. When packed up it is only 5 ft. by 3 in., so can be conveniently taken into the carriage or placed in the netting of the railway carriage.

